# Neonatal pain management practices in Somali region of Ethiopia: insights from neonatal intensive care unit providers

**DOI:** 10.3389/fped.2024.1344244

**Published:** 2024-02-02

**Authors:** Dawit Abebe, Afework Orcho, Jemberu Chane, Sinetibeb Mesfin, Wubareg Seifu

**Affiliations:** ^1^ School of Nursing and Midwifery, College of Health and Medical Sciences, Jigjiga University, Jigjiga, Ethiopia; ^2^ School of Public Health, Department of Epidemiology, College of Health and Medical Sciences, Jigjiga University, Jigjiga, Ethiopia; ^3^ School of Nursing and Midwifery, College of Health and Medical Sciences, Haramaya University, Harar, Ethiopia

**Keywords:** pain, neonatal pain, healthcare provider, Somali region, nursing

## Abstract

**Background:**

Neonates admitted to neonatal intensive care units experience an average of 8–17 moderate to severe painful procedures per day. Because neonates lack the cognitive capacity to express their pain's location or severity, they are very dependent on healthcare providers to recognize, assess, and manage their pain. The health and development of newborns are negatively impacted by persistent or untreated pain experienced early in life. Therefore, studying neonatal pain management practices and associated factors in healthcare is critical to tackling workforce problems, enhancing neonatal care, and lowering the long-term health impacts of neonates.

**Method:**

From January 1 to 30, 2023, a facility-based cross-sectional study design was used at six public hospitals in the Somali region of Ethiopia. A total of 336 healthcare providers enrolled using a simple random sample technique. A self-administered, structured questionnaire was utilized to collect the data. The analyses used bivariate and multivariate logistic regression. To find the association between the outcome and predictor factors, the odd ratio and the 95% CI were computed.

**Result:**

The study revealed that 35.4% [95% CI 30.4%–40.5%] of respondents reported that they had good neonatal pain management practices. Ever having undergone training in neonatal pain assessment and management [AOR = 2.26 (95% CI 1.259, 4.07)], availability of pain assessment tools [AOR = 3.05 (95% CI 1.249, 7.469)], and having a favorable attitude toward neonatal pain management practice [AOR = 3.71 (95% CI 1.525, 9.035)] were found to be factors with a significant association with neonatal pain management practice.

**Conclusion:**

Based on the study's findings, there is a low level of neonatal pain management practice among healthcare providers in the Somali region. The study emphasizes the significance of having access to pain assessment tools and the requirement for healthcare professionals to get training on neonatal pain assessment and management.

## Background

The International Association for the Study of Pain (IASP) currently defines pain as “An unpleasant sensory and emotional experience associated with actual or potential tissue damage or described in terms of such damage” (1). Late into gestation, the brain's pain pathways and pain perception center are well developed, and fetuses and neonates have the potential to recognize, perceive, and respond to painful stimuli (2).

Neonates admitted to neonatal intensive care units (NICUs) experience an average of 8–17 moderate to severe painful procedures per day (3, 4). Responses to pain in the neonate are associated with changes in behavior, physiology, and metabolism, and there are more than 40 pain assessment tools to gather information from each of these three classifications of pain responses, which are the pillars for pain management (5, 6).

In 1996, pain was recognized as the “fifth vital sign” that should be routinely monitored by healthcare providers in clinical practice (7). Because neonates lack the cognitive capacity to express their pain's location or severity, they are very dependent on healthcare providers to recognize, assess, and manage their pain (8, 9). Healthcare providers also must rely on frequent, accurate pain assessment tools and management guidelines; many healthcare providers have difficulties in implementing a neonatal pain assessment and management (10, 11).

Inevitably, newborn babies will undergo painful procedures as part of standard neonatal care. The health and development of newborns may be negatively impacted by persistent or untreated pain experienced early in life. Distinctly, they may cause alteration in brain development and function, poor motor function, intracranial hemorrhage, decreased immune response, and substantial long-term effects on neurosensory, cognitive, behavioral, pain processing, and health outcomes that can continue well through puberty and into adulthood (12–14).

Despite recommendations from the AAP and other experts, surveys continue to show that neonatal pain assessment and management remain inconsistent and insufficient during invasive procedures in NICUs (15). According to a review of the different available literature, a few studies from Sub-Saharan Africa have reported different levels of neonatal pain management practice (16, 17). Besides only a few studies conducted in Ethiopia, there has not been any research conducted outside of the capital city. Therefore, this study aimed to provide relatively strong and additional baseline evidence on neonatal pain management practice and associated factors of an under-researched region of Ethiopia's Somali region.

## Materials and methods

### Study setting, design, and period

The study was conducted at six public hospitals with NICU services in the Somali region from January 1 to 30, 2023. The Somali regional state is 1 of the 11 regional states in Ethiopia, located in the eastern part of the country 600 km from Addis Ababa, the capital city of Ethiopia. Based on 2012 Ethiopian Fiscal Year (EFY) figures from the Central Statistical Agency (CSA) of Ethiopia, 6.2 million people are estimated to live in the region; 86% of them live in rural areas, compared to 14% who live in cities, and 85% of them lead pastoral nomadic lifestyles (CSA 2017). There are 12 hospitals, 208 health centers, and 1,214 health posts in the region. This study was conducted among healthcare providers who had ever worked in the six public hospitals with NICU facilities in the Somali region, which were Jigjiga University Sheik Hassan Yabarre Referral Hospital (JJU-SHYRH), Karamara General Hospital (KH), Qabridahar Hospital (QH), Gode General Hospital (GGH), Degehabur Hospital (DH), and Warder Hospital (WH).

### Source population, study population, and eligibility criteria

All healthcare providers who had ever worked in public hospitals in the Somali region that provided NICU services comprised the source populations. All healthcare providers who had ever worked in the NICUs of each facility in the 2 years before the data collection period were included. The study excluded healthcare professionals who were on yearly, sick, or maternity leave and healthcare providers at free service at the time of the data collection.

### Sample size determination and sampling procedure

The sample size was calculated using the single population proportion formula with the following assumption: 95% confidence level, 5% margin of error, and 32.2% proportion of neonatal pain management practice, according to a recent study conducted in Addis Ababa, Ethiopia; after adding 10% contingency for the non-response rate, finally, 369 study participants were obtained. After the total number of healthcare providers who had ever worked in each of the selected public hospitals in the past 2 years before the data collection period (*N* = 429) was identified, the total sample size (*n* = 369) was proportionally allocated to each hospital. The study participants were selected by using simple random sampling, whereas the healthcare provider caring for the neonate who had participated in the observation was chosen through a convenience sample technique.

## Data collection tools and procedure

### Data collection instrument

Data were collected using a structured, pre-tested, and self-administered English version questionnaire. These structured questionnaires have different parts: Part One- Sociodemographic characteristics (5 Questions), Part Two- Organizational factors (5 Questions), and Part Three -Knowledge-related items (20 Questions), composed using the Likert scale, with the following responses: I disagree (1 point), I partially disagree (2 points), I do not know (3 points), I agree (4 points), and I strongly agree (5 points). These received a 1–5 rating. Then came Part Four-Attitude-related items (20 Questions), composed using the Likert scale, with the following responses: I disagree (1 point), I partially disagree (2 points), I do not know (3 points), I agree (4 points), and I strongly agree (5 points). These also received a 1–5 rating. Finally, there came Part Five- Practice items of healthcare providers toward neonatal pain management (18 Questions), with answers as “Yes” (1 point) and “No” (0 points). The questionnaires were extracted and adapted from different kinds of valid and reliable instruments used in previous literature with the same topics (4, 17–19).

### Data collection procedure

Six female midwives who had previous data collection experience were responsible for data collection. To supervise the data collectors and the data collection process, three supervisors (public health experts with a BSc degree) were chosen. The principal investigator spent 2 days training the supervisor and data collectors on the purpose of the study, the instruments' contents, how to select participants, how to fill out the questionnaire, and how to approach individuals ethically. All study participants had their understanding of the study's aim, the consent form, the confidentiality issue, and informed consent guaranteed. The overall activity during the data collection period was strictly supervised by the principal investigator and supervisors.

Preliminarily to the distribution of the self-administered questionnaire, a non-participant observation was undertaken on 10% of the total sample size. The self-administered questionnaire was disseminated to participants after their daily shifts but before they departed from the hospital. The duly completed questionnaires were subsequently gathered on the same day.

### Operational definitions

#### Adequate knowledge

The mean score of the respondent's knowledge questions was 70.2. Respondents who scored above the mean value were regarded as having adequate knowledge of neonatal pain management practice (19, 20).

#### Inadequate knowledge

The healthcare providers who scored below the mean value were regarded as having inadequate knowledge of neonatal pain management practice (19, 20).

#### Favorable attitude

The mean score of respondents for attitude-related items was 76. Respondents who scored above the mean value were regarded as having a favorable attitude.

#### Unfavorable attitude

The healthcare providers who scored below the mean value were regarded as having an unfavorable attitude toward neonatal pain management practice.

#### Good practice

The mean score of respondents for reported neonatal pain management practice-related questions was 11.1. Respondents who scored above the mean value were regarded as reporting good neonatal pain management practice (18, 20).

#### Poor practice

The healthcare providers who scored below the mean value were regarded as reporting poor neonatal pain management practice (18, 20).

#### Presence of analgesic

Availability of analgesic drug used for neonatal pain management in the NICU.

#### Presence of protocol/guideline

Refers to the existence of standard neonatal pain management guidelines in the NICU.

#### Presence of standard tool

Availability of neonatal pain assessment tool in the NICU.

### Data quality control

To further ensure the quality of the data collection tool, a pre-test was conducted on 5% (18 healthcare providers working at the NICU of Hiwot Fana Hospital) of the total calculated sample size 2 weeks before the actual data collection. The questionnaire was examined for its clarity, understandability, and simplicity. Following the pre-test, the questions were examined and restructured following the suggestions and remarks made by seniors. To ensure the accuracy of the data, the completed questionnaires were reviewed for accuracy. Both the principal investigator and the hired supervisors oversaw providing on-the-spot supportive supervision and a daily questionnaire review.

### Data processing and analysis

The data had been verified, coded, entered into EpiData version 3.1, and compared to the original data, and corrections were made as per the findings. After that, the data were exported to the Statistical Package for Social Science [SPSS] Version-27 software for analysis. Through SPSS's transform function, the variables were computed and recorded. Descriptive analysis was performed to compute proportions and summary measures. Tables, figures, simple frequency, and summary measures were employed to present the processed data.

The attitudes of healthcare providers were asked to score 20 questions on a five-point Likert scale related to neonatal pain management practice, a score of 1–5 was given according to their response, and items were then summed up out of 100. The mean score of the respondents for attitude was 76. Finally, those respondents who scored mean and above were labeled as having a favorable attitude toward neonatal pain management. In relation to knowledge related to neonatal pain, healthcare providers were asked to score 20 questions on a five-point Likert scale, a score of 1–5 was given according to their response, and items were then summed up out of 100. The mean score of respondent's knowledge questions was 70.2, and those respondents who scored mean and above were labeled as having adequate knowledge of neonatal pain management.

For neonatal pain management practice, which was computed by summing up all relevant 18 practice items, a score (No = 0 and Yes = 1) was given according to their response, and items were then summed up out of 18. The mean score was 11.1. Finally, those respondents who scored mean and above were labeled as having good neonatal pain management practice based on the report.

In the bivariate analysis, the crude odds ratio with 95% CI was estimated to determine the crude association between each independent variable with the dependent variable by using binary logistic regression. All variables with *P* < 0.25 at a 95% confidence level during the bivariate analysis were included in the multivariate analysis to control all possible confounders. By employing standard error, a multicollinearity test was performed to look for linear correlations between independent variables. A standard error greater than 2 was thought to be suggestive of multicollinearity. As a result, variables having standard errors greater than 2 were flagged for deletion. To assess the fitness of the models, the Hosmer–Lemeshow goodness-of-fit test was used. The omnibus test was significant, with a *p*-value < 0.05, which does not include the null value in the 95% CI, and it reported factors having a statistically significant association with neonatal pain management practice.

During the multivariate analysis, adjusted odds ratios with 95% CI were calculated to determine the factors associated with neonatal pain management practice. The independent variables were revealed to have a statistically significant association with neonatal pain management practice at the level of statistical significance *P* < 0.05 and without a null value in the 95% confidence interval.

## Results

### Socio-demographic characteristics of respondents

A response rate of 90.3% was achieved by including 336 out of the 372 healthcare providers who were sampled in the study. The study's participants ranged in age from 21 to 51 years, with a median age of 27 years, and 136 (40.5%) of the respondents fell within the 26–30 age group. Male respondents were the majority at 190 (56.5%). Regarding respondents' educational backgrounds, more than 50% of them (185, 55.1%) were found to have a BSc degree. More than one-third of respondents (37.2%) had experience working in the NICUs for 6 months, and nearly one-fourth of the respondents (80, 23.8%) had received any training on neonatal pain and neonatal pain management while working in the NICUs (Table 1).

**Table 1:** Socio-demographic characteristics of healthcare providers in public hospitals of Somali region, eastern Ethiopia, 2023.

Variables	Frequency (*n*)	Percent (10%)
Age group (in years)		
21–25	93	27.7
26–30	136	40.5
31–33	65	19.3
Above 35	42	12.5
Sex		
Female	190	56.5
Male	146	43.5
Educational level		
Diploma	89	26.5
BSc	185	55.1
MSc	23	6.8
MD	39	11.6
Work experience in NICU (in months)		
less than 6	125	37.2
6–12	71	21.1
12–24	87	25.9
Above 24	53	15.8
Receive any training on neonatal pain and neonatal pain management while being in NICU		
Yes	80	23.8
No	256	76.2

### Organizational factors

Out of 336 participant healthcare providers, 199 (59.2%) revealed the availability of pain assessment tools in the unit. A total of 193 (57.4%) participant healthcare providers declared the availability of protocols and guidelines for neonatal pain management. More than half (54.5%) of the respondents revealed that there were no available analgesics in their unit. Only 98 (47.9%) and 110 (59.8%) respondents claimed the availability of a pain management policy in place and the availability of support from their leadership on neonatal pain management, respectively (Table 2).

**Table 2:** Organizational factors related to neonatal pain management in public hospitals of Somali region, eastern Ethiopia, 2023.

Variables	Frequency (*n*)	Percent (%)
Pain assessment tools availability		
Yes	199	59.2
No	137	40.8
Protocols and guidelines for neonatal pain management availability		
Yes	193	57.4
No	143	42.6
Availability of analgesics in the unit		
Yes	153	45.5
No	183	54.5
Availability of pain management policy in place		
Yes	98	29.2
No	238	70.8
Availability support from leadership on neonatal pain management		
Yes	110	32.7
No	226	67.3

### Attitudes of healthcare providers toward neonatal pain management

Out of the total study participants, 59.2% [*n* = 199; 95% CI (53.9%–64.6%)] of the respondents had a favorable attitude toward neonatal pain management practice.

The study found that just 29 (8.6%) respondents firmly agreed that infants and children suffer the same level of pain as adults, whereas 149 respondents (44.3%), almost half, disagreed. Moreover, 159 (47.3%) participants claimed that neonates have the right to appropriate assessment and management of their pain, and 138 (41.1%) agreed that full treatment of neonatal pain is a humanitarian issue.

More than half (185, 55.1%) of the respondents agreed that the accurate judgment of a neonate's pain depends on their primary nurse, and 168 (50%) reported that the use of pain assessment tools for determining a neonate's pain leads to an appropriate method of pain relief. Only 69 (20.5%) respondents strongly agreed that the measurement and control of a neonate's pain can affect their healing process and reduce their hospital stay (Table 3).

**Table 3:** Attitude of healthcare providers toward neonatal pain Management in public hospitals of Somali region, eastern Ethiopia, 2023.

Items	Response									
	Disagree		Partially disagree		I don't know		Agree		Strongly agree	
	*N*	%	*N*	%	*N*	%	*N*	%	*N*	%
Neonates and children experience pain equal to that experienced by adults	149	44.3	33	9.8	42	12.5	83	24.7	29	8.6
Parents should not be present during painful procedures	69	20.5	57	17	68	20.2	120	35.7	22	6.5
Pain management and pain relief are of priority in neonate's treatment	21	6.3	31	9.2	25	7.4	189	56.3	70	20.8
Neonates have the right to appropriate assessment and management of their pain	25	7.4	18	5.4	20	6	159	47.3	114	33.9
The most accurate judge of the intensity of the neonate's pain is the their primary nurse	20	6	30	8.9	36	10.7	185	55.1	65	19.3
Full treatment of pain is a humanitarian issue	16	4.8	21	6.3	47	14	138	41.1	114	33.9
To better assess neonate pain, the nurse can discuss with their parents	30	8.9	27	8	32	9.5	182	54.2	65	19.3
Assessment and control of neonate pain lead to improving their parents’ satisfaction	27	8	20	6	30	8.9	179	53.3	80	23.8
Failure to assess and manage the neonate's pain affects their body and mind in the long term	30	8.9	26	7.7	34	10.1	177	52.7	69	20.5
The nurse's physical and mental fatigue can affect neonate pain relief	34	10.1	41	12.2	32	9.5	153	45.5	76	22.6
Like other vital signs, pain scores should be documented	27	8	50	14.9	37	11	133	39.6	89	26.5
To ensure the patient's comfort and pain relief is one of the most important tasks of nurses	23	6.8	47	14	35	10.4	143	42.6	88	26.2
Communicating with and educating neonate's parents play an effective role in relieving pain	25	7.4	43	12.8	26	7.7	172	52.1	67	19.9
Available tools for the measurement of pain are best for determining pain severity in neonates	30	8.9	56	16.7	45	13.4	147	43.8	58	17.3
When the necessary procedures have been done for the patient, the persistence of pain does not cause problems	66	19.6	84	25	46	13.7	109	32.4	31	9.2
Using pain assessment tools for determining neonate's pain leads to an appropriate method of pain relief	22	6.5	55	16.4	35	10.4	168	50	56	16.7
Measurement and control of a neonate's pain can affect the healing process and reduces the hospital stay	35	10.4	43	12.8	38	11.3	151	44.9	69	20.5
Evaluation and measurement of a neonate's pain should be considered as one of the vital signs when examining the neonate	20	6	38	11.3	32	9.5	185	55.1	61	18.2
Comparable stimuli in different people produce the same intensity of pain	78	23.2	51	15.2	41	12.2	136	40.5	30	8.9
Measurement and control of pain in a neonate leads to their improved quality of life	36	10.7	44	13.1	28	8.3	172	51.2	56	16.7

### Knowledge of nurses about neonatal pain management practice

Out of the total study participants, 65.8% [*n* = 221; 95% CI (60.4%–70.5%)] had good knowledge about neonatal pain management practice, while 34.2% had poor knowledge.

Out of the 336 participants, 150 (44.6%) and 154 (45.8%) of them agreed that both full-term and pre-term newborns experience pain, respectively. Only 113 respondents (33.6%) agreed that pain might impair a newborn's vital signs. Additionally, 145 (43.2%) respondents stated they were aware that noise and light influence a newborn's response to pain.

More than one-fourth of the respondents (88, 26.2%) strongly agreed that healthcare professionals fail to recognize the pain of newborns. Nearly one-third of those respondents (122, 36.3%) were aware that one of the vital signs in newborns is pain. Moreover, 158 (47%) in total said that newborn pain evaluation needs to be systematized.

Nearly half of the respondents (158, 47%) disagreed that submitting newborns to repeated painful procedures may have harmful effects on their development (Table 4).

**Table 4:** Knowledge of healthcare providers toward neonatal pain management in public hospitals of Somali region, eastern Ethiopia, 2023.

Response										
Items	Disagree		Partially disagree		I don't know		Agree		Strongly agree	
	*N*	%	*N*	%	*N*	%	*N*	%	*N*	%
Preterm newborns feel pain	29	8.6	19	5.7	43	12.8	154	45.8	91	27.1
Full-term newborns feel pain	28	8.3	22	6.5	45	13.4	150	44.6	91	27.1
Pain can affect a newborn's heart rate, respiratory rate, temperature, blood pressure, oxygen saturation, and intracranial pressure	88	26.2	56	16.7	48	14.3	113	33.6	31	9.2
Pain can affect a newborn's facial expressions, limb movements, and crying	28	8.3	51	15.2	37	11	142	42.3	78	23.2
Light and noise may affect a newborn's reactions to pain	31	9.2	17	5.1	55	16.4	145	43.2	88	26.2
A newborn's pain is not recognized by professionals	29	8.6	21	6.3	47	14	151	44.9	88	26.2
A newborn's pain is not considered by researchers	37	11	27	8	41	12.2	166	49.4	65	19.3
Newborns react to pain in a particular way	86	25.6	68	20.2	48	14.3	111	33	23	6.8
Pain is considered one of the vital signs in newborns	78	23.2	52	15.5	41	12.2	122	36.3	43	12.8
Pain assessment in newborns must be systematized	29	8.6	21	6.3	43	12.3	158	47	85	25.3
Pain assessment should be part of the nursing prescription	28	8.3	42	12.5	32	9.5	159	47.3	75	22.3
Newborns require analgesics due to the maturity of the nervous system to feel pain	40	11.9	43	12.8	40	11.9	156	46.4	57	17
Neonatal pain can be assessed without the use of scales	24	7.1	33	9.8	54	16.1	164	48.8	61	18.2
The use of scales for pain assessment is important to the practice	20	6	24	7.1	42	12.5	173	51.5	77	22.9
It is important to record pain on the newborn's chart	29	8.6	20	6	47	14	169	50.3	71	21.1
216. Recording pain assessment is a prerequisite to its control	22	6.5	25	7.4	48	14.3	122	36.3	119	35.4
217. Nurses have sufficient knowledge to assess pain in newborns	21	6.3	24	7.1	49	14.6	120	35.7	122	36.3
218. Pain management in newborns depends on its assessment	33	9.8	16	4.8	22	6.5	156	46.4	109	32.4
219. Recording a newborn's pain assessment will result in relief	31	9.2	37	11	23	6.8	193	57.4	52	15.5
220. Submitting newborns to repeat painful procedures may have harmful effects on their development	158	47	25	7.4	43	12.8	89	26.5	21	6.3

### Pain management practice

The participants were asked how they assessed the pain experienced by neonates. Out of the total of 336 participants, 257(83.3%) and 241 (76.5%) of them reported that they assessed a neonate's pain through crying and facial expressions, respectively.

Moreover, 242 (72%) reported that they encourage and use breastfeeding to relieve pain in newborns, and 221 (65.8%) reported that they encourage skin-to-skin contact to relieve the pain. Half (171, 50.8%) of the participants reported using pharmacological and non-pharmacological treatments combined to relieve pain in newborns (Table 5).

**Table 5:** Reported pain management practice of healthcare providers in public hospitals of Somali region, eastern Ethiopia, 2023.

Response				
Items	Yes		No	
	*N*	%	*N*	%
I assess newborn's pain through crying	280	83.3	56	16.7
I assess newborn's pain through facial expressions	257	76.5	79	23.5
I assess newborn's pain through body movement and agitation	166	49.4	170	50.6
I assess newborn's pain through the vital sign	237	70.5	99	29.5
I use scales to assess pain in newborns	154	45.8	182	54.2
I record newborns’ pain scores on their medical charts	195	58.0	141	42.0
I use non-nutritive suckling to relieve pain in newborns	181	53.9	155	46.1
I encourage breastfeeding to relieve the pain in newborns	242	72.0	94	28.0
I encourage skin-to-skin contact to relieve the pain in newborns	221	65.8	115	34.2
I offer oral glucose or sucrose to relieve newborn pain prior to painful procedures	166	49.4	170	50.6
I offer oral glucose or sucrose to relieve newborn pain during painful procedures	151	44.9	185	55.1
I position the newborn to relieve their pain	229	68.2	107	31.8
I perform facilitated tucking in newborns during painful procedures	196	58.3	140	41.7
I read pain management guideline	181	53.9	155	46.1
I use more than one non-pharmacological treatment to relieve the pain of newborns	202	60.1	134	39.9
I use pharmacological and non-pharmacological treatments combined to relieve pain in newborns	171	50.8	165	49.1
I avoid using the IM route in the administration of analgesics	220	65.5	116	34.5
I advise the mothers of the newborns to use non-pharmacological techniques along with pain medications	252	75.0	84	25.0

**Figure 1:**
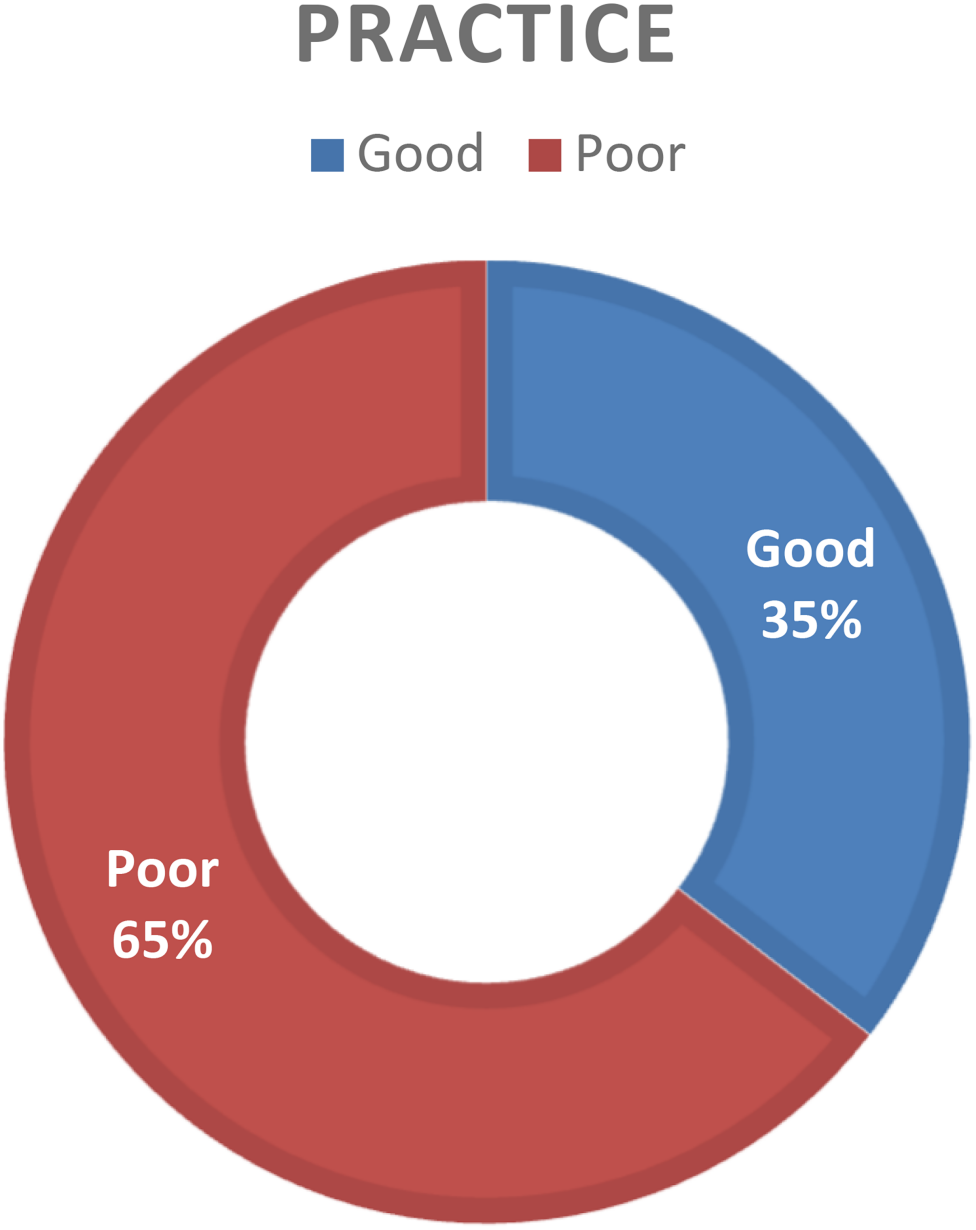
Categorized reported neonatal pain management practice among healthcare providers working in public hospitals of the Somali regional state, eastern Ethiopia, 2023.

Furthermore, 119 (35.4%) [95% CI 30.4%–40.5%] respondents reported that they had good neonatal pain management practices (Figure 1).

### Factors associated with neonatal pain management practice

In the bi-variable model, healthcare providers with over 12 months of work experience in the NICUs [COR = 1.99 (95% CI: 1.038, 3.819)], ever received training on neonatal pain assessment and management while in the NICUs [COR = 1.955 (95% CI: 1.172, 3.261)], availability of pain assessment tools [COR = 4.30 (95% CI: 2.55, 7.241)], availability of protocols and guidelines for neonatal pain management in the unit [COR = 3.028 (95% CI: 1.858, 4.93)], availability of analgesics in the unit [COR = 1.587(95% CI: 1.012, 2.490)], having a favorable attitude toward neonatal pain management practice [COR = 4.620 (95% CI: 2.72, 7.82)], and having good knowledge about neonatal pain management [COR =  2.175 (95% CI: 1.31, 3.597)] were found to have a statistically significant positive association with neonatal pain management practice.

To control confounding and to find independent factors of pain management practice, multivariable analysis was used. The following variables with a *p*-value of <0.25 in the bi-variable logistic regression analysis were candidates for multivariable analysis: Healthcare provider with over 12 months of work experience in the NICU, having undergone training on neonatal pain assessment and management, availability of pain assessment tools, availability of protocols and guidelines for neonatal pain management in the unit, availability of analgesics in the unit, having a favorable attitude toward neonatal pain management practice, and having good knowledge about neonatal pain management.

Out of those associated variables in bi-variable analysis, healthcare providers who had ever undergone training in neonatal pain assessment and management while in the NICU were two times more likely to practice neonatal pain management practice [AOR = 2.26 (95% CI: 1.259, 4.07)], those who claimed the availability of pain assessment tools in the NICU were three times more likely to perform neonatal pain management [AOR = 3.05 (95% CI: 1.249, 7.469)], and those with a favorable attitude toward neonatal pain management practice were almost four times more likely to practice neonatal pain management [AOR = 3.71 (95% CI: 1.525, 9.035)], thus maintaining their significant association with neonatal pain management practice in multivariable analysis (Table 6).

**Table 6:** Factors associated with neonatal pain management practice among healthcare providers toward neonatal pain management in public hospitals of Somali region, eastern Ethiopia, 2023.

Variable		Neonatal pain management practice				Bivariate logistic regression		Multivariate logistic regression
		Good		Poor		COR	P-V	AOR (95% CI)
		*N*	%	*N*	%			
Experience	<6 months	45	36	80	64	1		1
	6–12 month	23	67.6	48	32.4	.852	.461	.775 (.393, 1.526)
	12–24 months	23	73.6	64	26.4	.639	.113	.586 (.303, 1.134)
	>12 months	28	47.2	25	52.8	1.99	.090	1.864 (.908, 3.827)
Training	Yes	38	47.6	42	52.4	1.955	.006	**2.26** (**1.259, 4.07**)**
	No	81	31.6	175	78.4	1		1
Availability of tools	Yes	95	47.7	104	82.5	4.301	.014	**3.05** (**1.249, 7.469**)*
	No	24	17.5	113	52.3	1		1
Protocol guideline	Yes	88	45.6	105	54.4	3.028	.126	.473 (.182, 1.233)
	No	31	21.7	112	78.3	1		1
Availability of analgesics	Yes	63	41.1	90	58.9	1.587	.068	1.601 (.966, 2.652)
	No	56	30.6	127	69.4	1		1
Attitude	Favorable	96	48.2	103	51.8	1		1
	Unfavorable	23	16.8	114	83.2	4.620	.004	**3.71** (**1.525, 9.035**)**
Knowledge	Good	91	41.2	130	58.8	1		1
	Poor	28	24.3	87	75.7	2.175	.406	1.286 (.711, 2.326)

CI, Confidence interval; COR, crude odds ratio; AOR, adjusted odds ratio.

* *P*-value < 0.05.

** *P*-value < 0.005.

### Observational checklist findings on neonatal pain management in hospitals

An observational checklist was conducted in six public hospitals on 10% of the total sample size where the study was performed. In the study, 37 healthcare providers were observed. Only 8 (21.6%) of them checked neonatal pain before and after giving medicine, 6 (16%) used neonatal pain assessment tools to check neonatal pain, and 5 (13.5%) completely documented the information about neonatal pain. Out of the six hospitals, a pain assessment tool was available in only two (33.3%), namely, JJU-SHYRH and Gode General Hospital, at the time of data collection. Pain-relieving medications were available in five (83.3%) hospitals, and neonatal pain management guidelines were available in only two (33.3%).

In conclusion, the observational checklist added further support to the results of the self-administered questionnaire. The results indicate that some healthcare professionals utilized pain assessment tools and recorded data, but many did not. Only two hospitals had access to the neonatal pain management guidelines and possessed pain assessment tools. All hospitals, except for one, had access to pharmaceuticals for pain relief, but it is crucial to make sure they are utilized properly and in tandem with effective pain management techniques. These findings highlight the need for continued education and training for healthcare professionals in neonatal pain assessment and management.

## Discussion

In this study, neonatal pain management practice and factors associated with it among healthcare providers who had ever worked in NICUs in six public hospitals were investigated. In multivariable analysis, ever having undergone training in neonatal pain assessment and management, availability of pain assessment tools, and having a favorable attitude toward neonatal pain management practice were found to be factors significantly associated with neonatal pain management practice. Overall, 35.4%; 95% CI 30.4%–40.5% of the respondents reported that they had good neonatal pain management practice. This finding is almost similar to the finding of a study conducted in Addis Ababa showing 32.2% and a study conducted in Ambo in central Ethiopia showing 37.3% (18, 19). The findings of this study were lower than the findings of studies performed in Italy, showing 65%, and France, showing up to 77%, for major painful procedures (21, 22). Disparities in healthcare facilities and resources may be the root cause of this variation. France and Italy may have more advanced methods of treating pain than Ethiopia because they are developed countries with better groundwork and healthcare resources. The third possibility can be that the level of education and training of healthcare professionals involved in neonatal care can also have an impact. Healthcare providers in Ethiopia have limited access to specialized training or education on neonatal pain management.

Several independent variables were discovered to have statistically significant associations with the outcome variable in this study. The self-reported neonatal pain management practice level was utilized to determine factors associated with newborn pain management practice among healthcare providers. Neonatal pain management was more likely to be practiced by healthcare providers who had received training on neonatal pain management than those who had not received standard training. This finding aligns with those of studies conducted in Addis Ababa, Ambo, and Amiens University Hospital in France (18, 19, 23). This might be due to healthcare providers being more likely to be conversant with national or international guidelines and best practices for neonatal pain management practice if they have undergone training in it (23). Healthcare providers who have received this training are more likely to adhere to these guidelines, leading to more consistent and standardized pain management practices.

The unit's availability of pain assessment tools was positively associated with healthcare provider neonatal pain management practice. This finding is consistent with the findings of studies conducted in Rwanda and Iran (16, 24). The mere fact that these tools offer unbiased and standardized techniques for assessing and quantifying pain in neonates could be one reason for the favorable association between the availability of neonatal pain assessment tools in the unit and healthcare workers' use of neonatal pain management (25). When healthcare professionals have access to validated pain assessment tools, this helps them to precisely identify and quantify the pain experienced by neonates, enabling improved decision-making regarding pain management practice. Furthermore, the presence of neonatal pain assessment tools may indicate a higher level of commitment on the part of the healthcare facility to manage neonatal pain. This commitment may improve pain management practice by raising the understanding and awareness of the importance of neonatal pain management among healthcare providers.

The finding of the present study showed that healthcare providers with favorable attitudes also have a positive association with neonatal pain management practice. This is in line with the findings of a qualitative study conducted on factors in the implementation of neonatal pain management in Iran (26) and a study conducted in Rwanda (16). One reason why favorable attitudes of healthcare providers toward neonatal pain management are positively connected with their practices is that attitudes can influence behavior. If a healthcare provider has a positive attitude regarding neonatal pain management, they may prioritize pain management practice and be more motivated to provide their patients with effective pain relief. Furthermore, a favorable attitude toward neonatal pain management may indicate a greater understanding and appreciation of the importance of neonatal pain management.

### Strengths and limitations of the study

The research attempted to mitigate social desirability bias by ensuring the anonymity and confidentiality of the participants. Instead of merely using nurses who work in the NICUs, the present study included all healthcare professionals who had ever worked in the NICUs, which resulted in a bigger sample size.

Healthcare professionals may find it difficult to recall their pain management practice, especially since this study included healthcare professionals who had ever worked in the NICUs, and healthcare professionals who had previously worked in the NICUs may have had trouble accurately recalling their pain management techniques from previous experiences. There is also the aspect of social desirability bias related to the self-administrative questionnaire nature of the study.

## Conclusion

Based on this study's findings, there is a low level of neonatal pain management practice among healthcare providers in the Somali region. This study underlines the value of having access to neonatal pain assessment tools for measuring pain and the necessity for healthcare providers to receive training in the assessment and management of neonatal pain. Additionally, improving the quality of care provided to neonates requires a favorable attitude toward neonatal pain management strategies. With special emphasis on enhancing healthcare provider training and access to pain assessment tools, our findings can guide policy and practice in neonatal pain management in the Somali region of Ethiopia.

## Data Availability

The original contributions presented in the study are included in the article/Supplementary Material, further inquiries can be directed to the corresponding author.
